# Differential Expression of Inflammatory Cytokines and Stress Genes in Male and Female Mice in Response to a Lipopolysaccharide Challenge

**DOI:** 10.1371/journal.pone.0152289

**Published:** 2016-04-27

**Authors:** Ashleigh Everhardt Queen, Megan Moerdyk-Schauwecker, Leslie M. McKee, Larry J. Leamy, Yvette M. Huet

**Affiliations:** 1 William and Mary, Department of Kinesiology, Williamsburg, VA, United States of America; 2 University of Oregon, Department of Biology, Eugene, OR, United States of America; 3 University of North Carolina at Charlotte, Department of Kinesiology, Charlotte, NC, United States of America; 4 University of North Carolina at Charlotte, Department of Biology, Charlotte, NC, United States of America; University of Navarra School of Medicine and Center for Applied Medical Research (CIMA), SPAIN

## Abstract

**Background:**

Sex plays a key role in an individual’s immune response against pathogenic challenges such that females fare better when infected with certain pathogens. It is thought that sex hormones impact gene expression in immune cells and lead to sexually dimorphic responses to pathogens. We predicted that, in the presence of *E*. *coli* gram-negative lipopolysaccharide (LPS), there would be a sexually dimorphic response in proinflammatory cytokine production and acute phase stress gene expression and that these responses might vary among different mouse strains and times in a pattern opposite to that of body temperature associated with LPS-induced shock.

**Materials and Methods:**

Interleukin-6 (IL-6), macrophage inflammatory protein-Iβ (MIP-1β), tumor necrosis factor alpha (TNF-α) and interleukin-1β (IL-1β) as well as *beta-fibrinogen* (*Fgb*) and *metallothionein-1* (*Mt-1)* mRNA expression were measured at four time points (0, 2, 4 and 7 hours) after injection of *E*. *coli* LPS in mice from three inbred strains.

**Results:**

Statistical analysis using analyses of variance (ANOVAs) showed that the levels of the all six traits changed over time, generally peaking at 2 hours after LPS injection. *Mt-1*, *Fgb*, and IL-6 showed differences among strains, although these were time-specific. Sexual dimorphism was seen for *Fgb* and IL6, and was most pronounced at the latest time period (7 hours) where male levels exceeded those for females. Trends for all six cytokine/gene expression traits were negatively correlated with those for body temperatures.

**Discussion:**

The higher levels of expression of Fgb and IL6 in males compared with females are consistent with the greater vulnerability of males to infection and subsequent inflammation. Temperature appears to be a useful proxy for mortality in endotoxic shock, but sexual dimorphism in cytokine and stress gene expression levels may persist after an LPS challenge even if temperatures in the two sexes are similar and have begun to stabilize.

## Background

It has been well described that females typically exhibit a lower susceptibility to bacterial infectious diseases than males [1]. While the definitive mechanism underlying this disparity is not completely understood, it has been demonstrated that hormonal and epigenetic elements are contributing factors in this widely observed phenomenon [2]. Previous studies have shown that these contributing factors can effect immunological variations between males and females, as well as induce pathogen-specific metabolic responses [3, 4, 5]. Regardless of mechanism, sexually dimorphic inflammatory responses are a prominent feature in lipopolysaccharide (LPS)- induced endotoxic shock and other conditions such as depression, various cardiovascular diseases, and cancer progression [6].

Cytokines are key regulators of the immune response. During infection, cells respond and can become activated by molecules present on the surface of the bacterial pathogen known as pathogen associated molecular patterns (PAMPs). When immune cells with pattern recognition receptors (PRRs) bind their specific PAMP, the cells become activated and mount a defense against the pathogen [7]. Toll-like receptor 4 (TLR-4), found on macrophages and neutrophils, aids in this response by forming complexes with other proteins, such as myeloid differentiation protein-2 (MD-2), and cluster of differentiation 14 (CD14). This newly formed complex recognizes LPS on gram-negative bacteria, and produces chemical mediators in the form of cytokines [8] that produce a myriad of responses, including hypothermia and other inflammatory reactions [9]. The specific type of proinflammatory cytokines produced, e.g. IL-6, TNF-α MIP-1β, and IL-1β, as well as the level of which they are up- or down-regulated has been shown to be dependent on levels of gonadal steroid hormones [8, 9, 10, 11, 12, 13, 14]

Cytokines also trigger expression of certain genes in the liver that may react to LPS-exposure by producing proteins that modulate stress [15]. One such stress modulator is Metallothionein-I (Mt-1), a small protein expressed in hepatocytes that binds to heavy metals, such as cadmium. Mt-1 responds to various stressors, such bacterial infections and activates macrophages to produce certain proinflammatory cytokines [16, 17]. Another stress protein, Beta-fibri nogen (Fgb), is overexpressed in the presence of LPS. As an acute phase protein, it is produced quickly after liver cell receptors interact with cytokines in the blood and thus serves to amplify the immune function [18,19]. Fgb causes certain chemokines, such as MIP-1β, to be expressed by macrophages.

In this study, we assessed the levels of four inflammatory cytokines and the expression of two stress genes in male and female mice at several time points following injection of *E*.*coli* LPS. In particular, we investigated the levels of these six traits to determine whether a significant difference was exhibited between the sexes in response to the LPS challenge. Body temperatures of mice post-LPS-injection were measured in order to conclude whether trends throughout the various time periods would negatively associate with the levels of the expression traits, as suggested from previous hypothermia-response studies [9]. Finally, sexually dimorphic immune responses in mice have been shown to differ with variations in genetic background [20]. To account for potential differences in strain type, we examined temperatures and the expression trait levels in three different inbred mouse strains.

## Materials and Methods

### Animals

Sexually mature mice (8–12 weeks; 20–30 g) from three different inbred strains: CD-1 (Charles River Laboratories, Wilmington, MA), BALB/C, and C57BL6 (BL6; The Jackson Laboratory, Bar Harbor, ME) were used. All experiments reported in this study were performed in accordance with the guidelines established in the “Guide for the Care and Use of Laboratory Animals” as adapted and promulgated by the National Institutes of Health.

### Body Temperature

Body temperature was assessed in male and female mice from each of the three inbred strains (n = 10-12/strain; total = 35). All mice first were injected intraperitoneally with a non-lethal dose of *E*. *coli* LPS (5mg/kg, Sigma Chemical Company, St Louis, MO). Temperatures then were recorded with an implanted body temperature transponder (IPTT-300, Bio Medic Data Systems, Seaford DE), with readings measured using a DAS-5001 reader/programmer (Bio Medic Data Systems) from one to eight hours in one-hour time intervals.

### Tissue and Blood Harvesting

A different group of mice (N = 125) was used to assess levels of four cytokines (interleukin-6, IL-6; tumor necrosis factor alpha, (TNF-α; interleukin-1 beta, IL-1β; and macrophage inflammatory protein-1 beta, MIP-1β), and the expression of two stress genes, *Mt-1* and *Fgb*, in mice at each of four time points. Mice at 0 hours were not given LPS, but were sacrificed in order to evaluate baseline expression of the cytokines and stress genes. Mice in the other treatment groups were first injected intraperitoneally with *E*. *coli* LPS as was done in the temperature experiment, and sacrificed at 2, 4, and 7 hours after the injection. Blood was taken from each mouse after sacrifice and centrifuged at 16000xg and 4°C to isolate the plasma. In addition, the livers and spleens were removed from each mouse, flash frozen in liquid nitrogen, and stored at -80°C.

### RNA Isolation and Reverse Transcription

Total RNA was prepared by homogenizing a small sample of liver tissue (approximately 250mg) from each individual mouse in TRIZol solution (Gibco-BRL, Gaithersburg, MD). RNA was extracted using phenol chloroform extraction, precipitated with isopropanol and washed with 70% ethanol. The RNA pellet was dried, resuspended in molecular biology grade water, and stored -80°C. RNA was then treated with RQ1 RNase—Free Dnase according to the manufacturer’s protocol (Promega Corportation, Madison, WI, USA). Finally, RNA was reverse transcribed to cDNA using ImProm-II^™^ Reverse Transcriptase (Promega Corporation, Madison WI, USA), according to the anufacturer’s protocol.

### Primers

All primers were purchased from Integrated DNA technologies (Coralville, Iowa), and are listed forward:reverse.

*Mt-1*: TgT CCT CTA AgC gTC ACC AC: ggA ggT gCA CTT gCA gTT CTT

*Fgb*: Cag AAC CgT Cag gAT ggC AgC g: CCA CCA gTT gAg Agg CCC CgT

*Actb*: TgC gTg ACA TCA Aag AgA Ag: Cgg Atg TCA Acg TCA CAC TT

### Amplicons

Amplicons were made by polymerase chain reaction (PCR) on cDNA from the animals exposed to LPS, with primers specific for *Mt-1* and *Fgb*. *Actb* was used as a housekeeping gene to adjust for variation between individual animals. PCR was carried out using Ready-To-Go^™^ PCR tubes with a pre-made bead containing all required enzymes (GE Healthcare, Buckinghamshire, UK). To accomplish this, 1 μL of cDNA was added to a mixture of 22 μL of molecular biology grade water and 1 μL of 10 μM primers, both forward and reverse, for a final volume of 25 μL. Following PCR, the samples were separated using gel electrophoresis and visualized. The bands containing the product were cut out of the gels and purified using a Qiaquick^™^ Gel Extraction Kit (QIAGEN, Valencia, CA). Amplicon concentrations were determined based on optical density (260nm) and then amplicons were serially diluted.

### Real-Time qPCR

PCR was performed on an ABI 7500Fast Real Time PCR System. The reactions consisted of cDNA, 1 μL of 10 μM primer, both forward and reverse, 9.5 μL molecular biology grade water, 1μL of sample and 12.5 μL of QuaniTect SYBR Green^™^ PCR Kit (QIAGEN) for detection, for a total of 25 μL. In thermocycling, all samples began with 2 minutes at 50°C and 15 minutes at 95°C, followed by 40–50 cycles of denaturation at 95°C, annealing between 60–70°C (based on primer used) and extension at 72°C for 15-25s and fluorescence measurement at 80°C for 25s. The *β-actin* thermocycling protocol was previously established [21]. Following amplification, a melting curve was obtained for all samples. The melting points of *Actb*, *Fgb* and *Mt-1* are 83.3°C, 81.5°C, and 86.5°C, respectively.

### Enzyme Linked Immunoabsorbant Assay

Plasma samples obtained at 4 hours were examined using a Mult-Analyte ELISArray^™^ (SA Biosciences, Valencia, CA) kit to detect the presence of multiple inflammatory cytokines (IL-1α, IL-1β, IL-2, IL-4, IL-6, IL-10, IL-12, IL-17α, IFN-γ, TNF-α, G-CSF, GM-CSF). This was done to determine the presence of specific cytokines that should be examined in testing the two and seven hour samples. Plasma samples for the seven hour and two hour time points were examined using Single-Analyte ELISArray^™^ kits (SA Biosciences) specific to each cytokine (TNF-α, IL-1β, IL-6, MIP-1β). In brief, 50μL of sample was added to each well, allowed to incubate for 2 hours, and then washed. A detection antibody specific to the cytokine was then added and allowed to incubate for 1 hour before washing. Avidin-HRP, used to detect the presence of bound antibodies, was then added and incubated for 30 minutes. The plate was developed and read at 450nm in a plate reader (BioTek, Winooski, VT). The absorbance readings were then used to calculate the total concentration of cytokine based on the standard curve generated.

### Analysis Traits

For IL-6, MIP1β, TNF, and IL-1β at 4 hours, and also for Il-6 at 2 hours, concentrations were so high that they exceeded the standard curve values and could not be determined. The analysis of the cytokines/stress gene expression levels therefore was based on all four time periods for *Mt-1* and *Fgb*, three time periods for MIP1β, TNF, and IL-1β (0, 2, and 7 hours), and two time periods (0 and 7 hours) for Il-6. For this reason also, sample sizes varied among the traits from 61 for Il-6 to 123 for *Mt-1* (see Table 1 below).

**Table 1:** Basic statistics (sample sizes, means and standard deviations) for the six expression traits adjusted for the effects of sex, strain, and time and their interactions, and correlations between each pair of these traits and with temperature.

Trait	N	Mean	Stdev	Correlations					
				*Fgb*	IL-6	MIP-1β	TNF-α	IL-1β	Temp
*Mt-1*	123	2.42	0.64	0.30**	-0.03	0.11	-0.08	0.07	-0.52*
*Fgb*	115	5.24	0.74		-0.13	-0.05	0.19	-0.05	-0.69*
IL6	61	29.15	11.54			0.54**	-0.00	-0.03	-0.50
MIP-1β	90	18.19	3.28				0.25	0.09	-0.74*
TNF-α	95	5.49	1.09					0.02	-0.63*
IL-1β	91	2.36	3.66						-0.57*

* = *P* < 0.05

**P < 0.01

### Statistical Analysis

The distributions of *Mt-1*, *Fgb*, IL-6, MIP1β, TNF, and IL-1β were adjusted for sex, strain, and time effects (and their interactions). Because all were found to deviate significantly from normality outliers were removed, and the values of these traits transformed by raising them to the 0.2 (*Fgb*), 0.25 (IL-1β), 0.3 (IL-6), 0.4 (*Mt-1* and MIP-1β) and 0.5 power (TNF-α) to promote normality. After transformation, the basic statistics (means and standard deviations) were calculated for each of the traits, as well as correlations between each trait pair that we evaluated for significance using the false discovery rate procedure [22].

Three-way analyses of variance (ANOVAs) were used to test temperature and each of the six expression traits for the effects of sex, strain, and time and all four interactions among these three factors (sex x strain, sex x time, strain x time, and sex x strain x time). As the detection of sexually dimorphic effects was of primary interest in this experiment, planned comparisons were conducted to test for variations among males and females in each strain, time interval, and strain by time combination. For other comparisons of groups within main effects or interactions not involving sex, we used Tukey’s post-hoc *t*-tests [23].

Spearman nonparametric correlations of the mean temperature readings were calculated for mice in each of the sex, strain, and time (0, 2, 4, and 7 hours) combinations with the comparable means for each of the six cytokine/gene expression traits. We predicted that these correlations would be negative in sign, and therefore evaluated them for significance using 1-tailed *t*-tests.

## Results

### Temperature Changes

**Fig 1:**
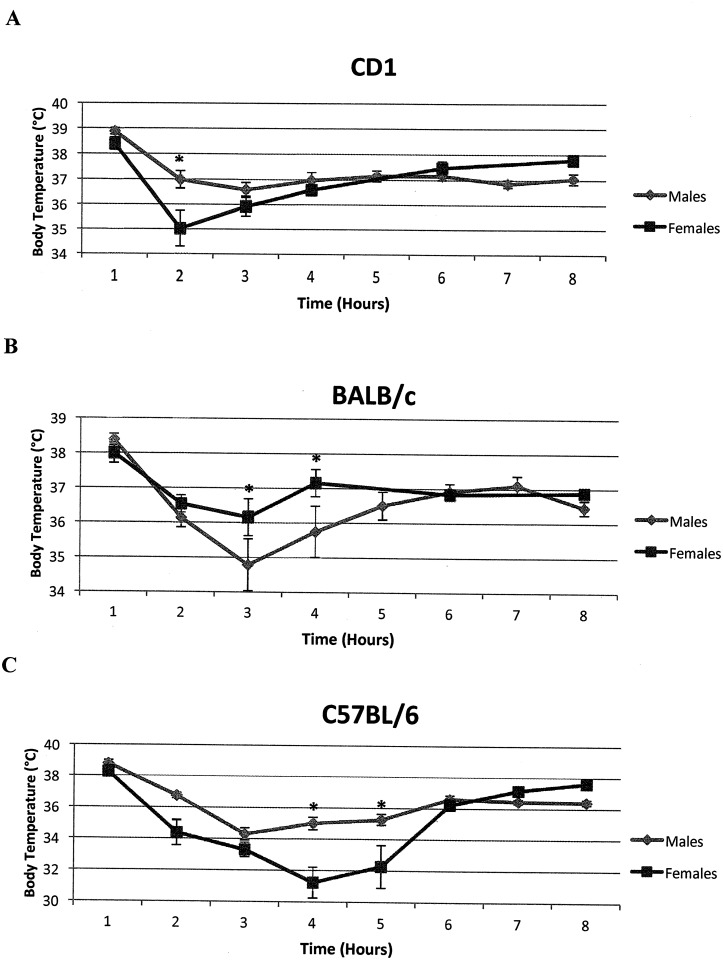
Body temperature changes over time following LPS exposure. Following a non-lethal dosage of 5mg/kg of LPS, mice were tested over a 9-hour time period for changes in body temperature. (Mean ± SEM) These were plotted by sex and strain. A. A significant difference in temperature was observed between male and female BALB/c mice at 3 hours and at 4 hours following LPS injection. B. A significant difference in temperature was observed between male and female CD1 mice at 2 hours, but not at any other time points measured. C. A significant difference was observed between male and female C57BL/6 mice at 4 and 5 hours following LPS injection. *P < 0.05.

Body temperatures in our sample of mice averaged 36.6°C, and throughout the sex, strain, and time combinations, varied from 29.6°C to 39.7°C. The three-way ANOVA for temperature showed significant (*F* = 9.63; d.f. = 1, 246; *P* = 0.002) effects for sex, but these effects varied among the strains and time intervals (sex x strain x time interaction: *F* = 3.41; d.f. = 12, 246; *P* = 0.0001). These differences are shown in Fig 1. Mice in all three strains show a mean temperature drop from the initial level, that reaches a low that varies from 2 to 4 hours after the LPS injection depending on the sex and strain. The planned comparisons also showed a significant difference in body temperature between the sexes at or near these points in time: at 2 hours for CD1 mice, 3–4 hours for BALB/c mice, and 2, 4 and 5 hours for BL6 mice. At these time points, CD1 and BL6 males have higher temperatures than females where the reverse is true for BALB/c males. The greatest drop in body temperature after the LPS injection is observed in the BL6 strain, whereas mice in the CD1 strain show the smallest drop.

### Basic Statistics

The basic statistics for the 6 expression traits, transformed as previously described and adjusted for potential effects of sex, strain, and time, are summarized in Table 1. The mean values for the traits range from 2.36 (IL-1β) to 29.15 (IL-6). The variability in these traits, as judged by the standard deviations relative to the means, was highest for IL-6 and lowest for *Fgb*. The correlations among these traits (after appropriate adjustment) were mostly low in magnitude, and two (*Mt-1* with *Fgb* and IL-6 with MIP1β) were statistically significant. Correlations of temperature with each of the six expressions traits all are negative, and all except for IL-6, are statistically significant. This result supports our expectation of a negative association between body temperature and the level of cytokine and/or stress gene expression.

### Expression Trait Differences

**Fig 2:**
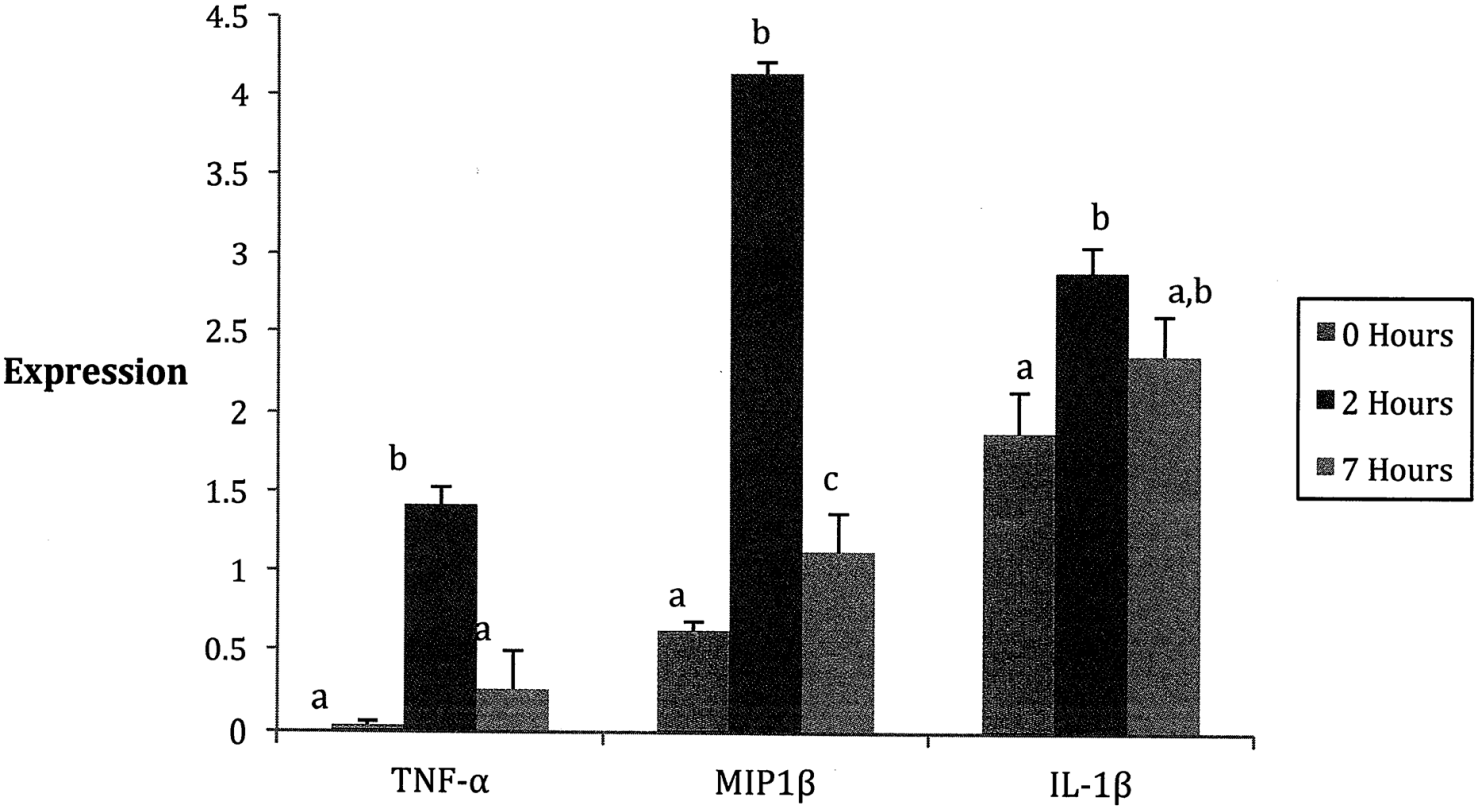
Time effect of cytokine expression. Concentration of serum cytokines observed from quantitative ELISAs. Due to transformations of all data, graphs are shown with arbitrary units. (Mean ± SEM) A. A significant difference was observed in the expression of MIP-1β, a chemokine that attracts macrophages, over the three time points tested. MIP-1β data was transformed by raising all values to 0.4. B. A significant difference was observed in expression of IL-1β, a proinflammatory cytokine, in the three time points tested. IL-1B, unlike others tested, did not show as high variation over the three time points. IL-1β data was transformed by raising all values to 0.25. C. A significant difference was observed in the expression of TNF-α, a proinflammatory cytokine, over the three time points tested. TNF-α data was transformed by raising all values to 0.5. Means with different superscripts within cytokine bars are significantly different. P < 0.05.

Overall changes in expression: In three-way ANOVAs, all six traits showed significant differences for time (*F* = 50.1–727.7; d.f. = 1–3, 60–99; *P* < 0.01–0.001), but for MIP-1β, IL-1β, and TNF-α, this was the only significant effect. The mean expression for these three traits increased from 0 to 2 hours following LPS injection, with post-hoc tests showing differences in the mean levels at these two time points were significant in all cases, but decreased (most significantly for MIP-1β) (Fig 2).

### Mt-1 specific interactions

**Fig 3:**
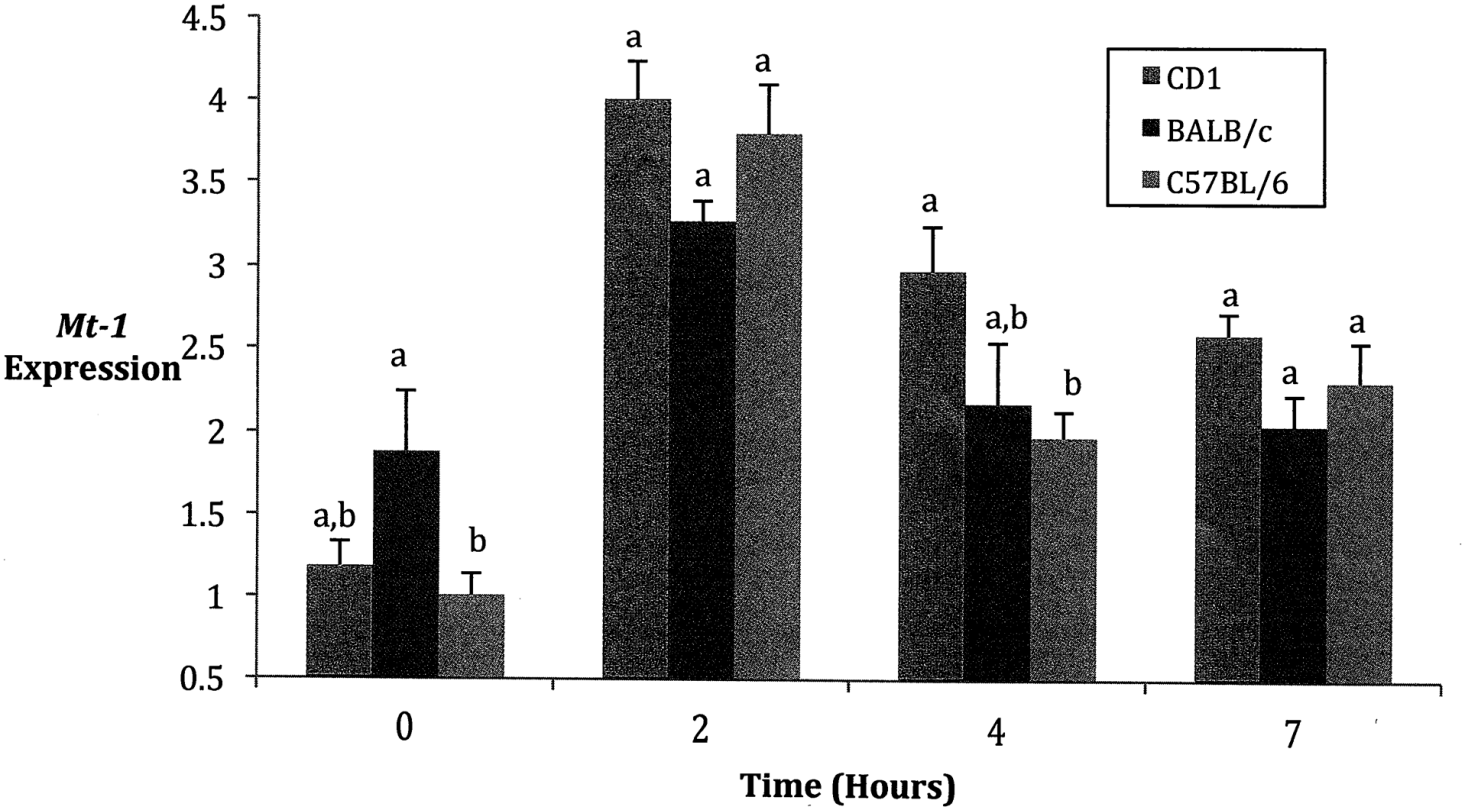
Effect of strain on the expression of metallothionein-1 in mice. Expression of Mt-1, a stress gene expressed in the liver, as compared to the control gene, Actb, following amplification with quantitative real time PCR. (Mean ± SEM) Mt-1 data was transformed by raising all values to 0.4. A. At 0 hours, a significant difference between BALB/c mice and C57BL/6 mice was observed. Neither was significantly different from CD1 mice in terms of Mt-1 expression. B. At 4 hours, a significant difference was observed from baseline levels for all strains. At this time, the levels were slightly higher for all three strains than the levels observed at 0 hours. F = 3.06; d.f. = 6,00; *P = 0.0 Means with different superscripts within time points are significantly different.

*Mt-1* showed a significant strain by time interaction in the ANOVA (*F* = 3.06; d.f. = 6, 99; *P* = 0.0087), with post-hoc tests revealing differences in expression levels between BALB/c and B6 mice at 0 hours, and between CD1 and B6 mice at 4 hours (Fig 3).

### Fgb specific interactions

**Fig 4:**
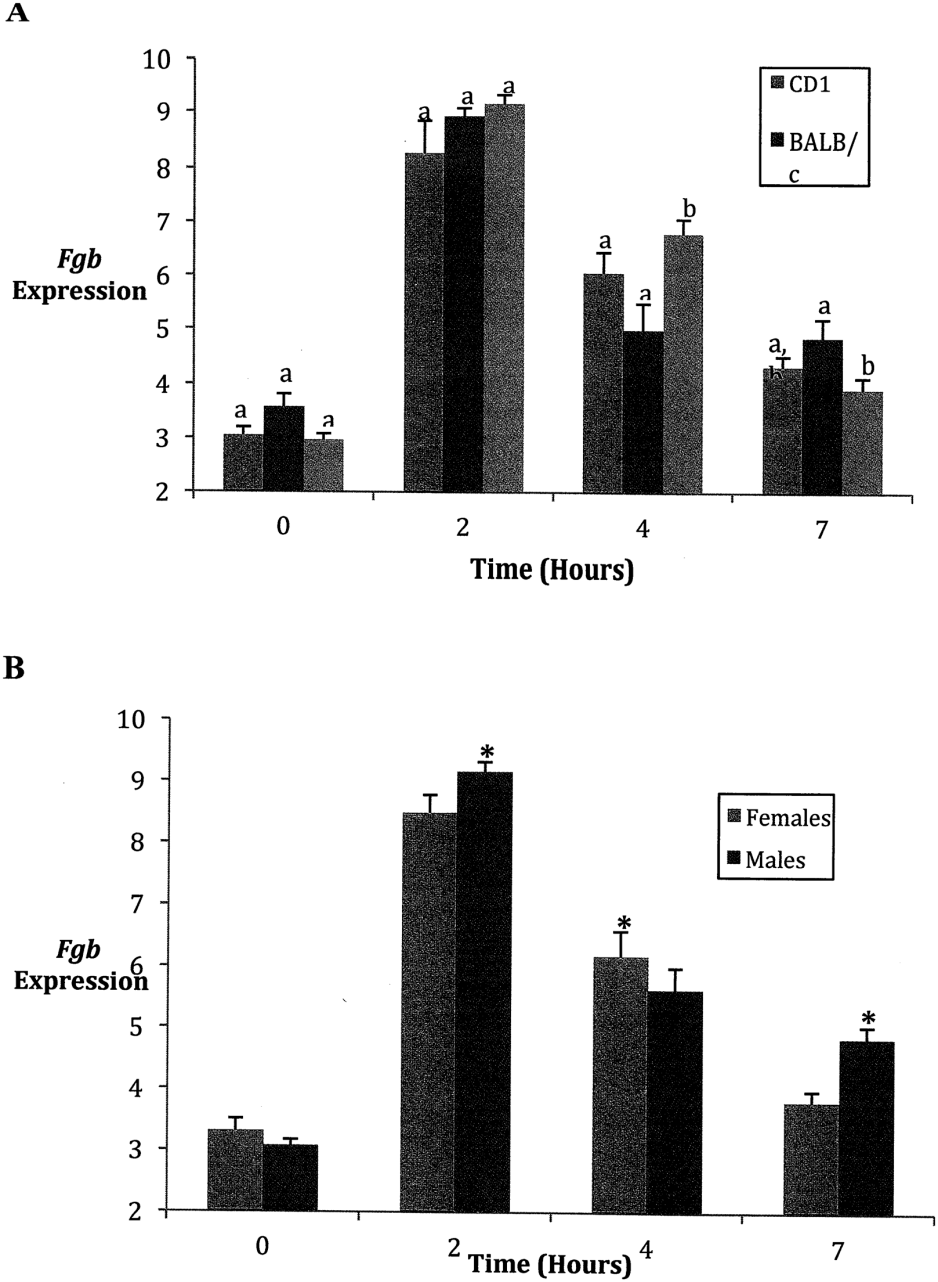
Beta-fibrinogen expression over time in three strains. Expression of Fgb mRNA as compared to control Actb mRNA following amplification of both genes with quantitative real time PCR. (Mean ± SEM) Fgb data was transformed by raising all values to 0.2. A. There was a significant strain by time interaction (*F* = 5.55; d.f. = 6, 91; *P* < 0.0001), with post-hoc tests showing differences in expression levels between BALB/c and B6 mice at 7 hours, and between BL6 and both other strains at 4 hours B. There was also a sex by time interaction (*F* = 6.73; d.f. = 3, 91; *P* = 0.0004)), with higher expression levels in males compared with females at the 2 hour and especially the 7 hour time periods, but the reverse at the 4 hour time period. Means with different superscripts are significantly different. P < 0.05.

*Fgb* also showed a significant strain by time interaction (*F* = 5.55; d.f. = 6, 91; *P* < 0.0001), with post-hoc tests showing differences in expression levels between BALB/c and B6 mice at 4 hours, and between BL and both other strains at 7 hours (Fig 4A). *Fgb* also showed a sex by time interaction (*F* = 6.73; d.f. = 3, 91; *P* = 0.0004)), with higher expression levels in males compared with females at the 2 hour and especially the 7 hour time periods, but the reverse at the 4 hour time period (Fig 4B).

### Il-6 specific interactions

**Fig 5:**
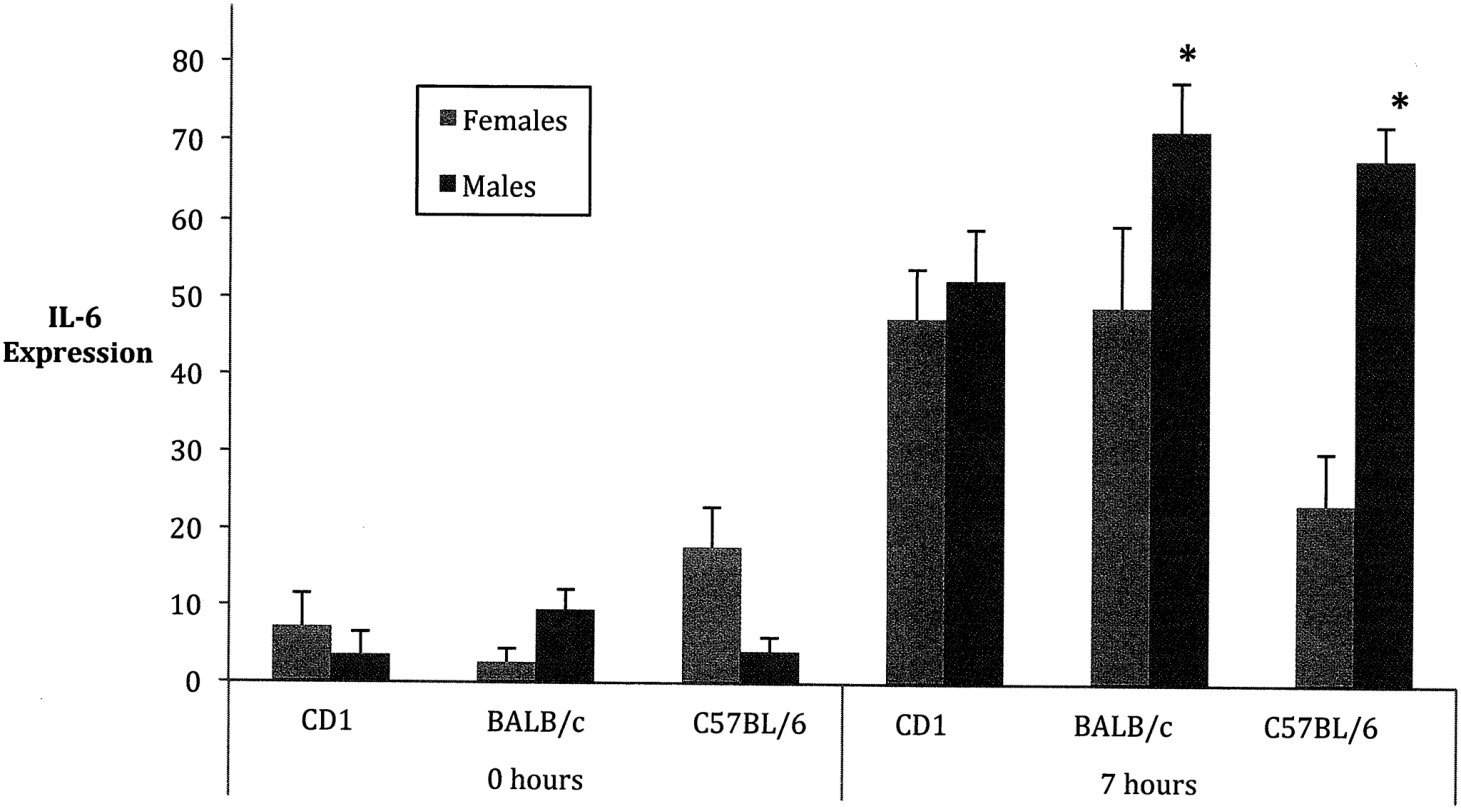
Effect of sex and strain on the expression of IL-6. Expression of serum IL-6, a proinflammatory cytokine, tested using quantitative ELISAs. (Mean ± SEM) IL-6 data was transformed by raising all values to 0.3. At 0 hours, a significant difference was observed, with any difference in sex or strain being dependent on the other variable. At 7 hours, a significant difference was observed, with any difference in sex or strain being dependent on the other variable. Differing letters indicate significance. P < 0.05.

IL-6 also showed a significant sex by strain by time interaction (*F* = 5.44; d.f. = 2, 49; *P* = 0.0074.), suggesting that sex differences for this cytokine vary and are dependent on the strain and time after LPS exposure. Fig 5 depicts the mean IL-6 values for male and female mice in each strain at the two time periods. The expression levels in males exceed those in females in the BALB/c and the BL6 strains, although only at the 7 hour time period.

## Discussion

Sexual dimorphism in response to bacterial infection has been well described. As early as the 1970’s, this phenomenon has been observed in hospital settings, where men exhibited a higher susceptibility to bacteremia than women [24]. It has also been found that women are less often admitted to the intensive care unit following septic infections than men [25]. One example is the sexual disparity witnessed in the infection rates of the opportunistic pathogen, *Vibrio vulnificus*. Pathogenesis resulting from infection with this bacterium often affects males more than females, with 80% of the mortality occurring in males [26, 27]. Sexual dimorphism was also observed in the current experiment as well, as observed in the response of the mice in the three strains to *E*. *coli*-LPS exposure. These dimorphic responses include differences between males and females body temperatures and for two (*Fgb* and IL-6) of the six cytokine/gene expression traits, however, these differences varied either among strains and/or the number of hours after exposure.

### Body Temperature

Body temperature was observed in our samples of mice as a proxy for mortality in endotoxic shock. As was shown, significant sexual dimorphism in body temperatures occurred at various times after LPS exposure for mice in the three strains. In two of the strains, CD1 and BL6, males showed higher temperatures than females. However, in the third strain, BALB/c, females exhibited a higher body temperature than the males. These differences between males and females were especially noticeable for mice in the BL6 strain, primarily because of the exceptionally low body temperature (mean = 31.2°C) for females 2 hours after LPS exposure. Temperature fluctuations were still moderate, however, with this lowest temperature maintained higher than 23.4°C, the level generally associated with endotoxic shock [28]. Nonetheless, our analysis suggests caution in attempting to predict body temperature effects from exposure to toxins such as LPS across different strains, sexes, or times after exposure.

As LPS exposure typically leads to hypothermia, we predicted that body temperature readings for mice in each sex and strain at the four time intervals after LPS exposure would be negatively associated with the expression levels of at least some of the six cytokine/stress gene traits. This was observed in 5 of these 6 traits, with only IL-6 (*r* = -0.50) being the exception. This correlation was nearly the same magnitude as that for *Mt-1* expression, with temperature (-0.52; *P* < 0.05), but, it did not reach significance. This was most likely due to the variations in sample sizes compared to previous experiments [13]. The parametric Pearson correlation of IL-6 with temperature was -0.66 and statistically significant. This provides strong evidence that the temperature patterns throughout the sex, strain, and times will negatively reflect the levels of all of the cytokine/stress gene expression traits.

### Trends in Cytokine Levels

TNF-α, a proinflammatory cytokine, and MIP-1β, a chemokine that directs movement of macrophages, were highly expressed at two hours following the injection of LPS, and returned to baseline values by seven hours. This occurred in all mice, regardless of sex or strain. At two hours, these proteins were at the highest level, coinciding with the time at which the temperature fluctuations were the greatest. This suggests that the release of these cytokines in response to LPS is associated with the drop in temperature. In addition, we found that peak MIP-1β and TNF-α expression levels both occurred at 2 hours, demonstrating a coordinated response by the immune system to LPS (Fig 2).

IL-1β also increased early and reached a high level by two hours, but unlike TNF-α and MIP-1β, it maintained higher levels following exposure to LPS (Fig 2). This initial expression may represent an early response to LPS that also corresponds to the decrease in body temperature; however, the return to normal body temperature did not produce decreased levels of this cytokine. Beyond the trends across time IL-1β levels did not express sexual dimorphism, contradicting previous studies which have demonstrated that levels of this cytokine in LPS-challenged murine-derived macrophages and human serum were higher in males than in females [13, 14].

Compared to the other cytokines, IL-6 appeared to exhibit a more prolonged response to pathogenic challenge. IL-6 was expressed at high levels in the mice at 7 hours after the LPS injection (Fig 5) although at this time temperatures had begun to return to normal and stabilize (Fig 1). Importantly, Il-6 displayed higher levels in males compared with females in 2 of the 3 strains, at this time point. Other studies also have reported higher levels of IL-6 in males compared with females following administration of LPS [6, 12, 29].

### Trends in Stress Gene Expression Levels

*Mt-1* is expressed in the liver and is an early responder to stressors such as LPS. In the current experiment, the highest *Mt-1* mRNA level occurred at two hours, declining after this time (Fig 3). *Mt-1* expression varied between strains at two time intervals, however, no observable differences where witnessed in levels between males and females among the strains and time intervals. Previous studies have observed sexual dimorphism in this cytokine, with significantly higher levels of Mt-1 in male compared with female rats fed from dams exposed to cadmium [30].

*Fgb* is a stress response gene that functions in the clotting pathway. This was exhibited at its high level two hours following the LPS challenge (Fig 4). At four hours, the level of *Fgb* expression in BL mice significantly exceeded that of expression in the other two strains. This presumably reflects the large decline in temperature shown by BL mice at this time (Fig 1). *Fgb* expression levels also were higher for males at 2 and 7 hours, but higher in females at 4 hours, however further studies may be required to determine the significance of this inconsistency. The probabilities from the planned contrasts for sex differences were both marginally significant: 0.0477 (2 hours) and 0.0497 (4 hours). The probability from this contrast at 7 hours, however, was much lower (0.0004). Thus just as was the case for Il-6, the major sexual dimorphism for *Fgb* expression occurred at 7 hours.

## Conclusions

Among the six traits analyzed, sexual dimorphism was observed in the expression of one cytokine and one stress gene. In both cases, sex differences were either time- (*Fgb*) or time- and strain-specific (IL-6). This sort of specificity may explain some of the conflicting results observed in previous studies regarding the presence and direction of sexual dimorphism for these types of traits following an LPS challenge. In general, the levels of these traits correlated with the temperature responses observed in the mice, reiterating that body temperature changes may serve as an appropriate proxy for endotoxic shock. Future studies may benefit from examining the associations of temperature changes with a broader range of cytokine and stress gene markers to gain a better understanding of those that are players in the strain- and sexually-dimorphic responses to endotoxins such as LPS. This will be important in determining which strains are better models for human responses.
